# Indications, types, and diagnostic implications of prenatal genetic testing in Sub-Saharan Africa: A descriptive study

**DOI:** 10.1371/journal.pone.0294409

**Published:** 2023-11-16

**Authors:** Abraham Fessehaye Sium, Tariku Shimels, Abdulfetah Abdulkadir Abdosh, Tesfaye Diress, Tigist Tsegaye, Tizibt Yifrashewa, Zewdu Terefework, Wondimu Gudu

**Affiliations:** 1 Department of obstetrics and Gynecology, St Paul’s Hospital Millennium Medical College, Addis Ababa, Ethiopia; 2 St. Paul’s Hospital Millennium Medical College, Addis Ababa, Ethiopia; 3 MRC-ET Advanced Laboratory, Addis Ababa, Ethiopia; Xuzhou Maternity and Child Health Care Hospital affiliated to Xuzhou Medical UniversityCare Hospital Affiliated to Xuzhou Medical University, CHINA

## Abstract

**Objective:**

To describe indications, test types, and results of prenatal diagnostic genetic amniocentesis among Ethiopian pregnant women.

**Methods:**

This study was a descriptive study on prenatal diagnostic genetic testing among Ethiopian pregnant women with certain indications and it was conducted at St. Paul’s Hospital Millennium Medical College (Addis Ababa, Ethiopia) from January 2017 to April 2023. Data on sociodemographic characteristics, genetic testing indications, types, and results were collected electronically. Data were analysed using SPSS version 23.

**Results:**

A total of 159 cases were analysed. The commonest indication for genetic testing among the study subjects was major fetal structural anomalies identified on specialized prenatal anatomic scanning of the index pregnancy detected in 71(44.7%) cases. Down syndrome and Edward syndrome were the commonest genetic aberrations detected accounting for 6.3% (10/159) and 4.4% (7/159), respectively. Among the rare genetic aberration detected were Di-George syndrome (0.6%) and Duchenne muscular dystrophy (0.6%).

**Conclusion:**

Findings of our study underscore the importance of diagnostic prenatal testing in a Sub-Saharan Africa setting, as common (trisomy 21&18) and rare genetic defects were identified using this important prenatal diagnostic testing. Considering the implications of detecting chromosomal abnormalities for future counselling and care, carrier state in parents for some chromosomal anomalies, and planning post-natal management of some abnormalities that are associated with aneuploidies (notably cardiac anomalies), initiation of diagnostic prenatal genetic testing service at tertiary public health facilities should be acted up on.

## 1. Introduction

Prenatal genetic testing is classified as invasive and non-invasive prenatal genetic testing (NIPT) [1]. NIPT has an advantage of low false positivity rate [1, 2]. Moreover, it can be used for screening at an earlier gestation. These advantages make it more preferable than the other invasive diagnostic testing procedures, which are chronic villus sampling (CVS) and genetic amniocentesis [3–5]. Serious genetic conditions such as: Trisomy 21 (Down syndrome), Trisomy 13 (Patau syndrome), Trisomy 18 (Edward syndrome), Monosomy X (Turner syndrome), and Klinefelter syndrome (XYY syndrome) carry high risk of miscarriage or infant mortality. Even if the babies survive it, there will be a long-term sequalae that will affect parents’ and child’s quality of life [6, 7]. Although NIPT is more preferrable than genetic amniocentesis or CVS for screening these serious conditions, the technical know-how about the testing and unavailability of skilled personnel make it inaccessible in low-income countries, compared to the invasive ones [8, 9].

Traditionally, fetal chromosome analysis has been performed using karyotyping with samples taken through chorionic villus sampling (CVS) or amniocentesis. However, this traditional method has a disadvantage of being time-consuming and labor intensive [10–12]. As of 2002, a Multiplex ligation-dependent probe amplification (MLPA) has appeared as an alternative molecular method of chromosomal analysis that allows the relative quantification of DNA sequences, has largely replaced traditional karyotyping as it can detect copy number variation in genomic sequences and be used to identify deletions and duplications at a submicroscopic level using as little as 50ng of DNA [13, 14]. A recent study shows that using specialized probe sets MLPA detects more chromosomal rearrangements, conferring significant risk of adverse outcome than karyotyping [15].

In General, genetic testing in Africa is limited to some academic centers or National Health Laboratory centers and it is mostly the invasive type. For example, in south Africa, there are well organized but small genetic services, based mostly in academic centers, provincial health departments and the National Health Laboratory Service [16].

Currently, there is scare literature regarding diagnostic prenatal genetic testing and patterns of chromosomal abnormalities in high-risk mothers in an African setting. In Ethiopia, there are no well-established prenatal genetic counselling and testing canters. In Ethiopia, clinicians have been using first-trimester combined screening to assign the genetic risk to the patients in the first trimester. Recently, prenatal diagnostic genetic testing (genetic amniocentesis) has been practiced at our Hospital, St. Paul’s Millennium Medical College (Addis Ababa, Ethiopia), in concert with a an in-country private genetic testing centre. Our study aimed to describe indications, test types, and results of prenatal genetic testing among Ethiopian pregnant women.

## 2. Methods and materials

### 2.1 Study design, setting, and population

This was a retrospective descriptive study on prenatal diagnostic genetic testing in a population of Ethiopian pregnant women and was conducted from May 5, 2023 to August 5, 2023. The study setting is St. Paul’s Hospital Millennium Medical College, is a tertiary leading referral hospital in Ethiopia for advanced patient diagnostics and treatment including advanced maternal-fetal medicine diagnostic and therapeutic interventions. It nests the biggest Maternal-fetal medicine center in Ethiopia, which is fully engaged in sub-specialty level care for Ethiopian pregnant women as well as delivering a high-quality sub-specialist training for fellows enrolled in the institution, who come from different parts of Ethiopia.

St. Paul’s Hospital Millennium Medical College (SPHMMC) has recently improved their ability to prenatally screen for and diagnose congenital anomalies and genetic diseases. This has been accomplished through training of maternal-fetal medicine (MFM) specialists with prenatal ultrasound expertise, genetic counselling education of nurses and availability of in-country diagnostic genetic testing (MRC-ET Advanced Laboratory, the only private genetic testing center in Ethiopia) able to detect pregnancies affected with aneuploidies and select microdeletion/microduplication syndromes [17]. In our hospital, both combined first-trimester and second-trimester screening test are provided prenatally to pregnant women and based on the results of this screening test and further genetic counselling and genetic amniocentesis is provided to those found to be at high-risk for genetic anomalies. In this study, all cases of pregnant women who had follow-up at our Hospital and a near-by private centre who had been managed by our MFM specialists and fellows and that had prenatal genetic test for specific indications: major structural anomalies or soft markers for aneuploidy on detailed ultrasound in the second or third trimester, high risk for aneuploidies labelled based on a combination of maternal risk factors, USG, and serum analytes, were retrospectively analyzed. The diagnostic genetic test was done exclusively on amniotic fluid samples obtained from genetic amniocentesis. MLPA (multiplex ligation-dependent probe amplification) PCR was used to perform all the genetic tests. No traditional karyotyping nor microarray nor sequencing was utilized in this study. No sample size calculation was applied. The exclusion criteria were those with incomplete data.

### 2.2 Data collection procedure

Data were extracted from amniocentesis registry at SPHMMC and genetic testing registry at MRC-ET Advanced Laboratory, where all the study subjects had their prenatal genetic testing, over a period of 6 years (from January 2017 till April 2023). The medical records of the study subjects were accessed from May 5, 2023, to June 5, 2023. Data on sociodemographic characteristics, genetic testing indications, types, and results were collected electronically, retrospectively. Non-random sampling technique was used during data collection, in which consecutive cases with complete data were included in the study.

### 2.3 Ethical consideration

A formal Ethical clearance letter was obtained for this study from St. Paul’s Hospital Millennium Medical College Institutional Review Board (IRB) on May 2, 2023. Data were fully anonymized before we accessed them and the IRB waived the requirement for informed consent. Hence, informed consent was not obtained from the study participants.

### 2.4 Data processing and analysis

Data were analysed using SPSS version 23. Simple descriptive analysis were used to analyse the data. Indications, test types, and chromosomal changes of prenatal genetic testing were analyzed. Frequency and proportions were used to present the results.

## 3. Results

Out of 186 pregnant women who had prenatal genetic testing, 159 were included in the final analysis after 27 cases were excluded due to incomplete data. The commonest indication for genetic amniocentesis and testing among the study subjects was major fetal structural anomalies identified on detailed sonography of the index pregnancy (Table 1). Seventy-one (44.7%) had major fetal structural anomaly as an indication for genetic testing, followed by another 21.4% (34/159) and 12.6% (20/159) who had prenatal genetic testing for high risk of Down syndrome and Edward syndrome respectively.

**Table 1:** Demographic characteristics, indication for genetic amniocentesis and diagnostic genetic test types for Ethiopian pregnant women who had prenatal genetic testing, 2017–2023.

Variable	Category	n		%	
Maternal age	Mean	30.2(19, 42)			
Place of antenatal care	Government hospital (SPHMMC)	97		61	
	Private practice	62		39	
Indications for Genetic testing	Major structural anomalies	71		44.7	
	High risk for Down Syndrome	34		21.4	
	High risk for Edward syndrome	20		12.6	
	Soft markers of aneuploidy (increased nuchal fold, choroid plexus cyst, cystic hygroma, and single umbilical artery)	18		11.3	
	Non-immune hydrops	4		2.5	
	IUGR	3		1.9	
	Prior child with muscular dystrophy (Duchene’s).	2		1.3	
	Others (unexplained elevated AFP, encephalomegaly, advanced maternal age, on maternal request, and history of CHD)	7		4.4	
Genetic test type	Chromosomal Analysis	103		64.8	
	Peripheral Chromosomal Analysis	20	12.6	
	Karyotype/trisomy	15	9.4	
	Trisomy/ Karyotyping/ Microdeletion syndrome	13	8.2	
	Others	5	3.1	
	Duchenne Muscular Dystrophy (DMD)	2	1.3	
	Turner syndrome	1	0.6	

From those who had major structural anomalies, 29 (18.2%) cases had fetal ventriculomegaly,11(6.9%) cases had multiple congenital anomalies, 8 (5%) cases had renal anomalies, and another 6(3.8%) fetal cardiac anomalies. Eighteen (11.3%) study subjects had their prenatal genetic testing for sonographic soft markers of aneuploidy ((increased nuchal fold, choroid plexus cyst, cystic hygroma, and single umbilical artery). Two pregnant women (1.3%) had history of previous affected child with Duchene’s muscular dystrophy as an indication for the prenatal genetic testing. Chromosomal analysis was the predominant genetic test type employed during the study period, accounting for 64.8%(103/159),followed by peripheral chromosomal analysis (12.6%, 20/159) and Karyotype/trisomy(9.4%, 15/159).

Analysis of genetic testing results showed that Down syndrome and Edward syndrome were the commonest genetic aberrations detected accounting for 6.3% (10/159) and 4.4% (7/159), respectively(Table 2). Patau syndrome, Di-George syndrome, Turner syndrome, and carrier for Duchenne muscular dystrophy were identified each in single pregnant woman representing each 0.63%.

**Table 2:** Amniotic fluid genetic testing results: Chromosomal changes for Ethiopian pregnant women who had prenatal genetic testing, 2017–2023.

Chromosomal changes (Abnormalities)	n	%
Normal (No change)	130	81.8
Trisomy 18(Edward syndrome)	10	6.3
Trisomy 21 (Down syndrome)	7	4.4
Trisomy 13(Patau syndrome)	1	0.63
Monosomy-X (Turner syndrome)	1	0.63
Carrier for Duchenne Muscular Dystrophy	1	0.63
Consistent with Di-George affected patients (Exons on the genes TBX5, exons 2, 3, 5, 8 and 9, GATA4 exons 5, 6 and 7)	1	0.63
Chromosome 10 (heterozygous duplication)	1	0.63
Ambiguous copy number changes of Congenital heart defect related genes	1	0.63
Others (Exon 3 of EVC and EVC2 genes, Exons 8–12 of PLOD1 gene located on chromosomal region 1p36, TBX1, Chromosomes 5 and 6, Chromosomes 22 and 4, and multiple chromosomes affected)	6	3.8

## 4. Discussion

In this study, the most common indications for prenatal genetic testing were sonographic findings of major fetal structural anomalies, high risk for trisomy 21 and 18, and findings of soft markers of fetal aneuploidy. The most frequently detected genetic aberrations were Trisomy 18(Edward syndrome), trisomy 21(Down syndrome). Among the rarest genetic abnormalities were Patau syndrome, Di-George syndrome, Turner syndrome, and carrier for Duchenne muscular dystrophy.

Genetic counselling has been shown to be an effective intervention in decreasing the negative impact of congenital anomalies and genetic conditions. However; this intervention is not available in most sub-Saharan Africa countries [18], and little is known regarding the practice of this testing in this region of Africa, compared to the developed part of Africa. Prenatal genetic diagnosis has been accessible at tertiary care levels across South Africa since the early 2000s [16]. An earlier study from Cameroon found a poor knowledge of genetic tests among medical students and physicians [19]. Another study that employed a face-to-face interview to study 130 parents with a living child affected with Sickle Cell Anemia demonstrated majority (89.8%) from these parents accepted the principle of prenatal genetic diagnosis for Sickle Cell Anemia [20]. A recent study on knowledge and attitude in genetic counselling in major hospitals in Addis Ababa (Ethiopia) demonstrated that there is increasing demand for genetic testing in Ethiopia [21]. Earlier, a survey study on patient preferences for prenatal testing including prenatal genetic testing, found that Ethiopian patients have interest in doing prenatal testing including prenatal genetic testing. Above 90% of the respondents in the study expressed their preference for prenatal genetic testing for 14 fetal conditions including trisomy 13, monosomy-x(Turner syndrome), and Duchenne muscular dystrophy(DMD)(11), which were also among the indications for prenatal genetic testing as well as confirmed fetal genetic defects with this testing in our study. Major structural fetal anomalies and findings of soft markers for aneuploidy preceded by high risk for trisomy 18 & 21(the most common chromosomal defects identified) were among the commonest indications for genetic testing in the present study.

Central nervous system malformations detected on ultrasound are strongly associated with and predictive of chromosomal abnormalities, especially trisomy 13 and 18 [22]. Among the major fetal structural anomalies, the most common indication for prenatal genetic testing in this study was fetal ventriculomegaly(close to 1 in 5 of the indications, 18.2%, were for this structural anomaly). Ideally, amniocentesis should be performed on all patients with fetal ventriculomegaly [23] and Karyotyping will identify chromosomal anomalies in 3–11% of the cases, most often trisomy 21 [24–26]. In fetuses with trisomy 21 and trisomy 18, cardiac anomalies are the most common structural sonographic features [27]. This evidence demonstrates the importance of having prenatal genetic testing in pregnancies complicated by major fetal structural anomalies.

Among the health problems that impose a heavy burden on families and a society in general are chromosome aneuploidies. The most common chromosome aneuploidies among fetuses are trisomy 21 (Down syndrome, T21), trisomy 18 (Edwards syndrome, T18), trisomy 13 (Patau syndrome, T13) [28]. From these, Down syndrome (DS) is the most commonly identified genetic form of mental retardation and the leading cause of specific birth defects and medical conditions [29]. In our study, this genetic defect was the commonest confirmed genetic disorder up on performing the genetic testing accounting for 6.3% (10/159) followed by Edward syndrome, which was identified in 7(4.4%) cases. There was only one case of Patau syndrome identified in this study. So were detected other rare genetic defects, namely Di-George syndrome, Turner syndrome, and carrier for Duchenne muscular dystrophy.

Although non-invasive prenatal genetic testing is being practiced worldwide, currently, invasive prenatal testing is still the most commonly practiced in low-income countries that includes Sub-Saharan Africa (SSA) [6]. Variations also exist in women’s knowledge of prenatal genetic testing s [30]. A US-based study found that African Americans and Latinos were less knowledgeable about genetic testing than Whites, and they were less likely to have the financial resources or insurance coverage that would facilitate access to the testing [31]. Full coverage of the cost of clinical genetic testing is not always available through public or private insurance programs, or a public healthcare system [32], which is also true in Ethiopia. Meeting the high patients’ preferences for prenatal genetic testing with expansion of prenatal genetic testing service, as discussed above, can be an effective strategy to address congenital genetic defects in Ethiopia and beyond across the Sub-Saharan Africa. However, the issue of affordability should be parallelly addressed for a wider scale of access. It has been stated that diagnostic genetic testing in SSA deserves increased attention as testing platforms become more affordable [21]. This should be balanced with availability of an option of accessing it at a cheaper price for patients with limited resources, to ensure greater expansion of this testing service across the region.

We recommend initiation of diagnostic prenatal genetic testing service at a tertiary public health facility with Maternal-fetal medicine sup-speciality care in Sub-Saharan Setting including Ethiopia, for two reasons. One, considering the importance of detecting chromosomal abnormalities in the index pregnancy for future counselling and care, and the fact that it will open a window for testing carrier state in parents for some chromosomal anomalies. Secondly, it’s importance neonatal screening and planning for management of some abnormalities that are associated with aneuploidies (notably cardiac anomalies).

Strengths of our study include being among the first studies from Africa to document the indications, test types, and test finding of prenatal genetic testing. The main limitations of this study are missing analysis of management approaches (pregnancy termination vs conservative management) and birth outcomes for those that had confirmed diagnosis of genetic defects. Retrospective data collection and lack of cost-benefit analysis are the other limitation of our study.

In conclusion, our study substantiates the practicality of diagnostic prenatal testing in a Sub-Saharan Africa setting with further evidence. It shows common (trisomy 21&18) and rare genetic defects (such as Di-George syndrome and Duchene’s Muscular dystrophy) were identified by sending amniocentesis samples for prenatal genetic testing at the private center after proper screening and evaluation by our MFM experts, though the final management outcomes are lacking. We recommend further prospective studies inclusive of cost-benefit analysis of invasive prenatal genetic testing, with a goal of making it affordable for pregnant women with limited resources and at high risk of fetal genetic diseases.
